# Cryptic Contamination and Phylogenetic Nonsense

**DOI:** 10.1371/journal.pone.0002316

**Published:** 2008-05-28

**Authors:** Anna Linderholm, Helena Malmström, Kerstin Lidén, Gunilla Holmlund, Anders Götherström

**Affiliations:** 1 Archaeological Research laboratory, Stockholm University, Stockholm, Sweden; 2 Department of Evolutionary Biology, Uppsala University, Uppsala, Sweden; 3 Department of Forensic Genetics and Forensic Toxicology, National Board of Forensic Medicine, Linköping, Sweden; University of Canterbury, New Zealand

## Abstract

Ancient human DNA has been treated cautiously ever since the problems related to this type of material were exposed in the early 1990s, but as sequential genetic data from ancient specimens have been key components in several evolutionary and ecological studies, interest in ancient human DNA is on the increase again. It is especially tempting to approach archaeological and anthropological questions through this type of material, but DNA from ancient human tissue is notoriously complicated to work with due to the risk of contamination with modern human DNA. Various ways of authenticating results based on ancient human DNA have been developed to circumvent the problems. One commonly used method is to predict what the contamination is expected to look like and then test whether the ancient human DNA fulfils this prediction. If it does, the results are rejected as contamination, while if it does not, they are often considered authentic. We show here that human contamination in ancient material may well deviate from local allele frequencies or the distributions to be found among the laboratory workers and archaeologists. We conclude that it is not reliable to authenticate ancient human DNA solely by showing that it is different from what would be expected from people who have handled the material.

## Introduction

New technologies for working with ancient DNA have increased knowledge and explanatory power in several disciplines bordering on evolution and genetics, and the addressing of anthropological issues through ancient DNA has aroused especial interest. It should be noted that the first major study exploring the power of ancient DNA was concerned with human remains [1], and that studies of ancient human DNA are still attracting a lot of attention after more than 20 years [2]. Ancient human DNA is challenging to work with, however. This was already recognized a decade ago when it was found that modern contamination is present to a high extent in reagents and materials [3], [4]. Even more alarming is the evidence that there may be higher concentrations of contaminating DNA than of authentic ancient DNA in specimens from museums and collections [5]. Furthermore, the contaminating DNA appears to degrade in a fashion similar to ancient DNA, making it hard to use damage patterns to discriminate between the two [6]. The only quantifiable difference to emerge to date is the level of DNA fragmentation [7], [8], which has proved useful when authenticating DNA from ancient hominids [9].

A list of tests and criteria aimed at establishing the authenticity of ancient DNA results was published in 2000 [10], and these criteria are often applied in ancient DNA studies. DNA results based on ancient human remains remain controversial in spite of meeting these criteria, however, the best illustration perhaps being the discussion that followed the publication of DNA sequences for the Italian Cro-Magnon remains [11], [12]. Thus, one criterion in particular has frequently been used in recent studies of DNA from ancient humans, that stating that sequences generated from the ancient specimens should “make phylogenetic sense”. This criterion was developed to authenticate DNA results obtained from non-human remains, i.e. it makes phylogenetic sense when a DNA sequence from a mammoth clusters with sequences from elephants rather than with human sequences. Initially this criterion was used to test the authenticity of an alleged dinosaur sequence [13] which proved to be a human sequence [14]. This criterion is nevertheless applied in an inverse fashion when studying DNA from ancient human remains. Haplotype and genotype frequencies in the geographical area where the remains are processed are assessed initially and all the laboratory technicians and archaeologists involved are also typed. The ancient specimens are then analysed and the data generated are accepted as being authentic if they deviate significantly from that initial background assessment [2], [15], [16], [17]. It should be pointed out that this mode of authentication is applicable only if every possible source of contamination can be accounted for.

To test this criterion, we selected a genetic marker that has a clear distribution pattern in Sweden. In a modern Swedish population 74% are carriers of a mutation that makes it possible to drink milk as an adult [18]. This high frequency should make it possible to trace any contamination arriving from the surroundings and the people who have handled the material. Next we selected some prehistoric skeletal material from Sweden to study the occurrence of the contamination often found in ancient DNA analyses. We extracted and typed a set of Swedish Neolithic human remains and negative controls for a genetic marker with globally varying allele frequencies in three laboratories and investigated whether the deviating results from the laboratories could be explained by contamination with local alleles or whether there were alternative explanations for the contamination.

## Materials and Methods

**Figure 1:**
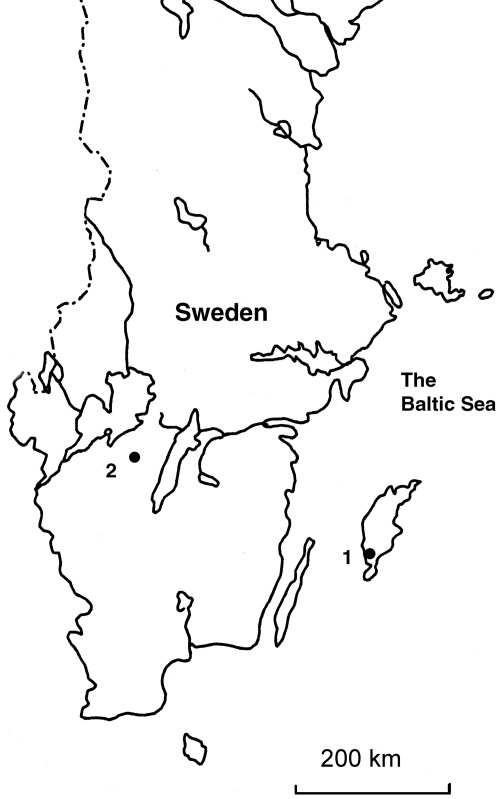
Map of Southern and Central Sweden showing the two archaeological sites referred to, 1. Gökhem, 2. Ajvide.

The human material originated from two Swedish archaeological sites, Ajvide on the Baltic island of Gotland, and Gökhem a mainland site in southern Sweden (Fig. 1). Both sites represent the middle Neolithic, spanning from 3300 to 2500 BC.

Initially we had access to 74 Neolithic human remains and 29 associated non-human specimens for which we already had ancient mitochondrial DNA data. These samples had been decontaminated using various techniques such as bleach pre-treatment prior to extraction of the bone powder and authenticated with real-time PCR quantification (mtDNA fragment of 80bp nt4542-4621, including the primer annealing sites). The measures followed a previously published protocol [8] in which the samples were cleaned with bleach, HCl and water, after which the outer surface was removed. About 100 mg of powder from each sample was further soaked in a 0.5% bleach solution for 15 minutes and then washed three times with water prior to the commencement of extraction. The water used was from different companies in all three laboratories, and the extraction method was a modification of silica spin column extraction [19], [20]. DNA was eluted in 90 to 100 µl aliquots of water or TE buffer.

**Figure 2:**
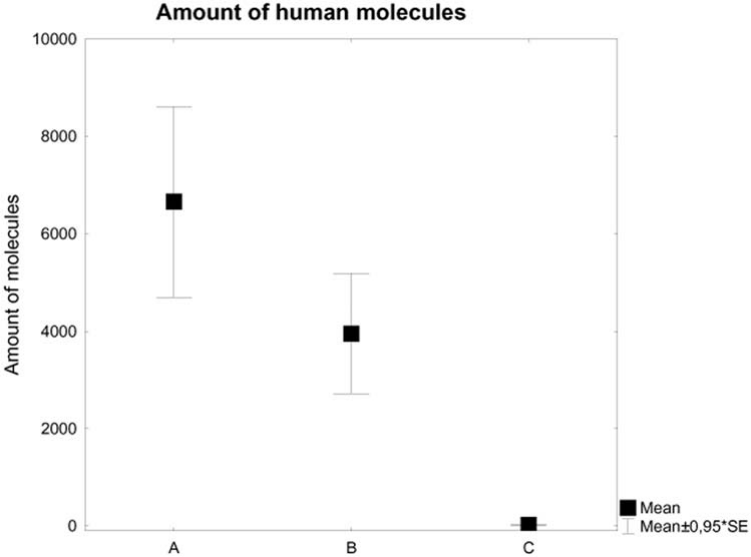
Presence of an 80bp mtDNA fragment in the 74 screened samples, the seven selected samples and 29 non-human samples. The subset of humans was selected from a quantitative pre-screening. Samples yielding sufficient DNA and containing sufficient bone material for repeated re-extraction, and originating from more than one collection were selected for further processing. Note that the same extracts that was used for the mitochondrial pre-screening was used for the -13910 typing in Linköping, while new extracts were produced in Stockholm/Uppsala. A = Human mitochondrial content in all human samples pre-screened for mitochondrial DNA, B = Human mitochondrial content in selected human samples and C = Human mitochondrial content in non-human samples pre-screened for human mitochondrial DNA.

We selected seven of the 74 human samples that had yielded significantly more human mitochondrial DNA (>1264 copies/5 µl extract) than the non-human samples (<197 copies/5 µl extract) (Fig. 2, Tab. 1), so that these human samples contained on average 198 times more human copies of the mitochondrial fragment tested for (3951±1296 SE) than the 29 non-human specimens (20±7.4 SE). The human samples were then decontaminated and extracted in two laboratories, Linköping (two independent extractions per sample) and Stockholm (a single extraction per sample), the separate extractions being carried out as independent replications performed by different people in each laboratory. Various negative controls were processed in parallel (a minimum of 18 and 4 non-human specimens and 16 and 3 extraction blanks in the Linköping and Stockholm laboratories respectively). DNA amplification was further performed in three laboratories, Linköping, Stockholm and Uppsala. The samples extracted in the Linköping laboratory were also amplified there, while the samples extracted in Stockholm were divided into two aliquots and further processed in Stockholm and Uppsala. All three laboratories are ancient DNA laboratories properly designed according to previously described standards [3], [10], with airlocks, positive airflow and ceiling UV radiation at night. All three laboratories are located in areas separated from any work with high quality DNA or post-PCR procedures, and the laboratory workers wore full zip suites, facemasks and several layers of gloves when in the laboratories.

Human specific primers (ordered separately for each laboratory from TAG, Copenhagen on three occasions) amplifying a 53 bp fragment were designed manually from a reference sequence (AY220757) where the biotinylated forward primer (5′→3′ GCTGGCAATACAGATAAGATAATG) and the reverse primer (5′→3′ GAGGAGAGTTCCTTTGAGGC) target a single nucleotide polymorphism (SNP) situated 13910 bp upstream of the LCT gene. Two PCR amplification protocols were used. In Uppsala 5 µl of extract was used in a 25 µl reaction, containing 2 mM MgCl_2_, 0.2 µM of each primer, 400 µM dNTPs and 2.5U Taq Polymerase (HotStarTaq, Qiagen), whereas in Linköping and Stockholm 5 µl of extract and 300 nM of each primer was added to PCR beads (Illustra Hot Start Mix RTG) in a 25 µl reaction. The PCR bead kit from Illustra was used in both the Linköping and Stockholm laboratories, with new batches used for each PCR amplification setup. The cycling conditions in Stockholm and Linköping (conditions for Uppsala are given in brackets when they deviate) were 95°C for 15 min (or 10 min), followed by 43 (or 47) cycles at 94°C for 30 s, 54°C(or 52°C) for 30 s, 72°C for 30 s, and a final extension at 72°C for 15 min (or 7 min).

The amplification was investigated on a 2% agarose gel in Stockholm and Uppsala and samples yielding a visible result were pyrosequenced^TM^ on a PSQ™ 96MA sequencer. All the amplified samples from Linköping were pyrosequenced without any verification, as they had been selected from samples known to yield DNA. Pyrosequencing is a real-time sequencing method in which the DNA sequence is identified from light emitted via an enzymatic reaction when bases are incorporated into the DNA molecule [21], [22]. In Linköping the PCR products were not checked on a gel prior to SNP typing. A commercial SNP reagent kit (Biotage, Uppsala) was used to examine the PCR products produced in all three laboratories, but different batches were used in each laboratory. In this step 25 µl PCR product from the samples was prepared according to the manufacturer's instructions. The sequencing primer was designed to anneal next to the SNP (5′→3′ CCTTTGAGGCCAGGG). Nucleotide dispensation was automatically retrieved using the PSQ™ 96MA SNP software (Biotage, Uppsala). The SNPs were scored automatically, edited using the PSQ™ 96MA SNP software and finally checked manually.

The results from Linköping were based on multiple (3-6) amplifications from two independent extractions, while those from Stockholm/Uppsala were based on single extractions followed by multiple (n = 3) and single amplifications respectively. Each amplicon from the negative controls was treated as an independent observation. The Mann-Whitney U test was used to compare the alleles observed between the datasets. As the results in Stockholm and Uppsala originated from the same extractions, they were pooled. Negative controls were compared with the human samples in both datasets, and the numbers of negative controls that yielded positive results for human samples, i.e. contaminated blanks, were then compared between Linköping and Stockholm/Uppsala. Negative PCR controls were not included in the analyses. All the statistical analyses were performed on STATISTICA 7.

## Results and Discussion

The seven human samples all yielded PCR products with identifiable SNPs in all three laboratories (Table 1). The four non-human samples and the three extraction controls processed in Uppsala and Stockholm were amplified 28 times, and all yielded amplicons with identifiable SNPs (Table 2). The 29 non-human samples and the 25 extraction controls processed in Linköping were amplified 84 times altogether, and yielded 8 amplicons with identifiable SNPs (Table 2).

**Table 1:** Amount of an 80bp mitochondrial fragment (number of templates per real-time PCR reaction, with 5 µl DNA extracts used in 25 µl reactions, average from two real-time amplifications), and allele status in seven samples of ancient human remains typed in three laboratories (Linköping, Stockholm, and Uppsala).

Sample	No. of molecules	Allele distribution for the LCT gene at Stockholm/Uppsala	Allele distribution for the LCT gene at Linköping
Ajv5	1263	C	C
Ajv14	2659	C	C/T
Ajv54	8714	C/T	C
Gök1	1395	C	T
Gök2	8988	C	C
Gök6	1389	C	C/T
Gök7	3249	C/T	C/T
T Freq		0.14	0.36

The allele status in the Linköping samples was based on between 3 and 7 amplifications deriving from two independent extractions, while 3 amplifications deriving from one extraction in each case were made in Stockholm and one in Uppsala.

**Table 2:** The non-human samples and negative extraction controls were amplified 84 times in Linköping and 28 times in Stockholm/Uppsala, producing 8 amplicons in Linköping and 28 in Stockholm/Uppsala.

Samples	Linköping			Stockholm/Uppsala		
	No. PCRs	Contaminated	Freq C	No. PCRs	Contaminated	Freq C
Non-human	43	4	0.75	16	16	0.156
Extraction blank	41	4	1	12	12	0.125
PCR blank	39	6	0.7	12	6	0.17

The frequencies of the contaminating C-allele are given.

**Figure 3:**
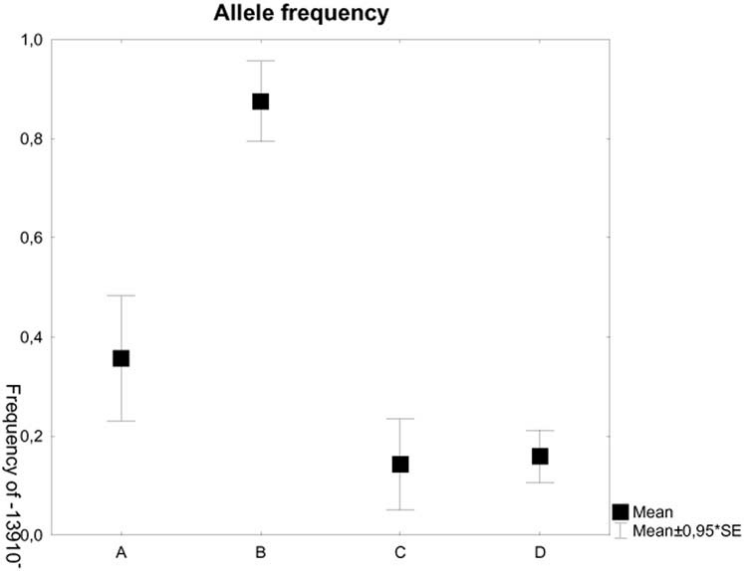
Frequency of the T allele located 13910bp upstream of the LCT gene. The groups illustrated are the human samples processed in Linköping, the human samples processed in Stockholm/Uppsala, the negative controls containing human DNA from Linköping, and the negative controls containing human DNA from Stockholm/Uppsala. A = Human samples processed in Linköping, B = Non-human samples processed in Linköping, C = Human samples processed in Stockholm/Uppsala and D = Non-human samples processed in Stockholm/Uppsala.

An obvious discrepancy was detected between the results from the three laboratories, the allele frequency for the derived allele (-13910T) in the seven human samples being 0.36 for Linköping and 0.14 for Uppsala and Stockholm (Fig. 3). The frequency of the -13910T allele in modern Sweden is 74% (21), whereas the only published results obtained for archaeological material showed no individuals carrying the -13910T allele in the eight Neolithic samples from Central Europe (16). In this case we observed a difference in allele frequencies in the contaminated samples by contrast with the frequencies expected on the basis of the modern distribution in the country where the material came from and was analysed.

We assume that the dataset generated in Linköping is more likely to be authentic than that generated in Stockholm/Uppsala, for three reasons. First, we have quantitative mitochondrial DNA data for the samples processed in Linköping (Fig. 2) showing that there is sufficient human DNA in the human samples and significantly more than in the negative controls, to provide for authentic results. This argument is generally accepted on its own as sufficient for authentication [23]. Second, significantly fewer negative controls were contaminated with modern human DNA at Linköping than at Stockholm and Uppsala (p = <0.001, Z = 7.149479 in a MWU test). Finally, there was a significant difference in allele frequencies between the negative controls that showed human results and the results obtained from the ancient human samples (p = 0.016, Z = −2.41109, Fig. 3) among those processed in Linköping. The seven human samples had a frequency of 0.36 for the derived allele while the contaminated controls (four out of 43 amplicons from non-human samples and four out of 41 amplicons from extraction controls) had a frequency of 0.88 for the same allele. There is no such difference between the negative controls and the ancient human samples in the results generated in Stockholm and Uppsala (p = 0.93, Z = −0.090854, Fig. 3).

The most striking result, however, is that the contaminating allele (-13910C) present in most of the negative controls and equally many of the samples processed in Stockholm and Uppsala is an allele that is as rare in Scandinavia as in the rest of Europe. None of the laboratory workers who had been closest to the material was a homozygote for the allele appearing in the contamination (AL, HM, and AG, although AG is a heterozygote). Furthermore, the material originates from two collections, making it improbable that one physical anthropologist with a deviating allele could have contaminated all the samples. If this had been the case, there would not have been any discrepancy between the results from Linköping and the other two laboratories. The origin of the contamination could lie in reagents manufactured outside of Europe, for example, and used in the various laboratories. Several of the reagents used in all three laboratories were from the same suppliers but from different batches and with different production dates. The water and the ethanol used in the laboratories were of different origins, and while PCR reagents from the same supplier were used in Stockholm and Linköping, PCR reagents from a different supplier were used in Uppsala (but note that the results from Uppsala did not deviate from those from Stockholm). Contamination problems with PCR reagents have been demonstrated in earlier studies [24]. Other possible sources could be the plastic ware or the gloves, for which the three laboratories use different suppliers.

More important, the contamination we detected in this material is of a type that we would not have expected from samples excavated and processed in Scandinavia, as the allele present in the negative controls is rare in this area (but note that the allele frequencies in the contaminated samples are similar to those known in parts of Asia, where much of the plastic ware and gloves had been produced). Thus our data raise concerns on how the authentication of ancient human DNA is currently taking place. It is not appropriate to authenticate results solely on the basis of deviation from the modern population or the scientists and laboratory workers who have been in contact with the material. This criterion is not sufficient on its own, but it could possibly be used in combination with other criteria such as real-time qPCR, typing of human DNA in a sufficiently large body of non-human material and massive amplicon cloning.
